# The Contribution of Mosaic Variants to Autism Spectrum Disorder

**DOI:** 10.1371/journal.pgen.1006245

**Published:** 2016-09-15

**Authors:** Donald Freed, Jonathan Pevsner

**Affiliations:** 1 Program in Biochemistry, Cellular and Molecular Biology, Johns Hopkins School of Medicine, Baltimore, Maryland, United States of America; 2 Department of Neurology, Kennedy Krieger Institute, Maryland, United States of America; 3 Department of Psychiatry and Behavioral Sciences, Johns Hopkins School of Medicine, Baltimore, Maryland, United States of America; University of Pennsylvania, UNITED STATES

## Abstract

*De novo* mutation is highly implicated in autism spectrum disorder (ASD). However, the contribution of post-zygotic mutation to ASD is poorly characterized. We performed both exome sequencing of paired samples and analysis of *de novo* variants from whole-exome sequencing of 2,388 families. While we find little evidence for tissue-specific mosaic mutation, multi-tissue post-zygotic mutation (i.e. mosaicism) is frequent, with detectable mosaic variation comprising 5.4% of all *de novo* mutations. We identify three mosaic missense and likely-gene disrupting mutations in genes previously implicated in ASD (*KMT2C*, *NCKAP1*, and *MYH10*) in probands but none in siblings. We find a strong ascertainment bias for mosaic mutations in probands relative to their unaffected siblings (p = 0.003). We build a model of *de novo* variation incorporating mosaic variants and errors in classification of mosaic status and from this model we estimate that 33% of mosaic mutations in probands contribute to 5.1% of simplex ASD diagnoses (95% credible interval 1.3% to 8.9%). Our results indicate a contributory role for multi-tissue mosaic mutation in some individuals with an ASD diagnosis.

## Author Summary

Recent sequencing experiments have shown that genetic mutations present in children but not their parents contribute to autism diagnoses in a large fraction of affected families. Here we address the question of whether mutations occurring uniquely in the children arise in the parents’ sperm or egg, or as mosaics in the child after conception. Using a dataset of 2,388 families, we find that while these mutations are primarily inherited from parental germ cells, 5.4% of these mutations appear to arise after conception. Mosaic mutations occur more frequently in probands relative to their unaffected siblings and from this enrichment we estimate that mosaic mutations contribute to 5.1% of autism diagnoses. In addition, we show that brain-specific mutations are not frequently detectable in individuals with an autism diagnosis. Our results indicate that some fraction of identical twins is expected to be discordant for an autism diagnosis due to genetic factors (post-zygotic mutation).

## Introduction

DNA is constantly exposed to natural and artificial mutagenic processes and therefore continually develops lesions and undergoes subsequent error-prone repair. In multi-cellular organisms, these mutations may arise at any time during development resulting in diverse organismal and cellular phenotypes, including disease. The severity of these phenotypes is dependent upon not only the particular genetic change but also the affected cell type and time in development at which the mutation occurs. Obligatory somatic disorders, in which prenatally lethal germline mutations occur post-zygotically, are one extreme [1].

In contrast to obligatory somatic mutation, *de novo* mutation is thought to primarily occur in the parental germline, typically resulting in genetic variation that is heterozygous in every cell of an organism. Such mutation is *de novo* in the sense that it is below the limit of detection in a parental sample (usually DNA derived from blood). An early report using comparative genomic hybridization indicated that large *de novo* copy-number variants are enriched in ASD probands [2]. From these results it was hypothesized, and subsequent microarray and whole-exome sequencing experiments have shown, that a substantial fraction of genetic liability arises *de novo* in every generation [3–15].

The exact developmental time at which *de novo* mutations occur however, is under active investigation. Some *de novo* variants discovered though whole-exome sequencing have properties consistent with mosaicism [8,11,16,17]. Recent experiments using high-depth targeted sequencing have indicated that eight of 27 likely causal variants in individuals with cortical malformations are present as mosaics, occasionally at very low alternate-allele read fractions (AARF) [18]. Mosaic mutations have been found to occur in single individuals of monozygotic twin pairs [19,20]. Furthermore, 6.5% of identified *de novo* mutations in individuals with severe intellectual disability occur as mosaics [21]. Here we show that *de novo* variation in a large whole-exome sequencing dataset is frequently mosaic and that such mosaic variation is likely to contribute to disease diagnoses in some affected individuals.

## Results

### Tissue-specific mosaic mutation

Obligatory somatic mutations typically occur in a localized fashion in tissues that share a common developmental origin. To examine the contribution of tissue-specific mutations in ASD, we generated whole-exome sequence data from paired postmortem frontal cortex (n = 16) and heart (n = 14) or kidney (n = 2) samples from individuals diagnosed with ASD (n = 12) and controls (n = 4). Sequence data were generated using the Illumina HiSeq platform with an average sequence depth of 95x across capture targets (S1 and S2 Tables). Variants were detected using the somatic variant callers Strelka and Mutect [22,23]. These programs detect mutations unique to single tissues from paired samples. Analyzing frontal cortex/heart or frontal cortex/kidney pairs resulted in the identification of 373 mosaic variants in the 32 samples. However, validation experiments indicated that all potential mutation loci were homozygous for the reference allele (*i*.*e*. all mutations chosen for validation were false positives; S1 Fig and S3–S6 Tables). Our findings agree with previous results that tissue-specific mosaic mutation in brain rarely occurs at the level of detection afforded by standard whole-exome sequencing experiments [24].

### Detection of mosaic mutations from single samples

In next-generation sequencing data, reads supporting the alternate allele at variant sites are known to be under-represented due to biases against non-reference alleles [25]. Further, many variant callers explicitly assume a diploid model. Therefore, the extent to which existing germline variant callers accurately genotype mosaic mutations in unpaired samples is uncertain. To evaluate our ability to discover mosaic mutation occurring in single samples, we obtained the Illumina Platinum Genomes sequence including NA12878, an individual for whom a high-confidence callset is available (ERP001960) [26]. We then characterize the sensitivity of the GATK HaplotypeCaller through *in silico* mixture experiments (S2 Fig). For this experiment, we utilized 200x sequence data from the Illumina Platinum Genomes (ERP002490; See Materials and Methods). Sequence reads from NA12878 were mixed with sequence reads from her son NA12882 over regions known to harbor variants from the high-confidence GIAB callset. Mixtures were then subsampled to depths of 30x and 50x with random fractions of reads from N12878 and NA12882. Variants were then called from the mixture and the sensitivity of the variant caller was assessed for variants known to be present in NA12878 but not NA12882. Sensitivity was also assessed with different values of–ploidy argument which alters the expected AARFs of heterozygous variants. These results demonstrated that higher–ploidy settings improved sensitivity for low frequency mosaic variants at the cost of higher memory usage and longer runtimes (S3 Fig).

### Mosaic mutations in the Simons Simplex Collection

Given that mosaic variants may be identified with germline variant callers, we sought to determine the mosaic status of variants in the Simons Simplex Collection (SSC), a large collection of simplex autism pedigrees [27]. Extensive phenotypic data and whole-exome sequence data have been generated for all members of the collection and two non-overlapping callsets have been generated from the SSC exomes [13,15]. To increase sensitivity for detection of mosaic variants we performed a complete re-calling of all samples in the SSC with a -ploidy 5 setting (S4 Fig). Variant filtration was performed using the GATK’s variant quality score recalibration (VQSR) pipeline and *de novo* variants were identified using find_denovo with default parameters. This resulted in the identification of 6,408 *de novo* variants, of which 3,355 and 228 were present in the Iossifov or Krumm callsets, respectively and 2,825 were unique to our callset (S7 Table). Average coverage of high quality sequence reads at positions with identified *de novo* variants was 94.6, indicating that mosaic variants are likely to be accurately detected, when they occur. Of variants identified by Iossifov *et al*. or Krumm *et al*. but excluded from our callset the majority were filtered by VQSR (S8 Table). The complete set of variants in the Iossifov or Krumm callsets, annotated with their inclusion or reason for exclusion from the current callset are listed in S9 and S10 Tables. We excluded fifteen families who had a child with more than 10 *de novo* mutations since these likely occur due to technical artifacts. After exclusion of these families, our callset contained 4,909 *de novo* variants. Variant effects were annotated using SnpEff and mosaic variants were identified from a binomial test with false-discovery protection using the Benjamini-Hochberg procedure [28]. This resulted in the identification of 1,036 mosaic variants at an FDR of 5%. Hereafter, we will refer to the variants identified as *de novo* but not mosaic as germline *de novo* variants.

To ensure that the variants in our callset were present in the samples, we performed Sanger sequencing of 97 (47 mosaic and 50 germline *de novo*) variants (Table 1, pre-filter). For all of our validation methods, we present both “detection precision” and “classification precision”, where applicable. We define “detection precision” as our precision for the presence of the variant in the sample, while we define “classification precision” as our precision for the presence of the variant in the sample and correct classification of the variant as either mosaic or germline *de novo*. Of the 97 reactions, sequencing was informative for 76. The variant of interest was identified in 100% of samples when the variant was annotated as germline *de novo*. However, in samples harboring a mosaic variant, precision for the presence of the variant was modest (54%). Mosaic variants that failed validation were often called with few reads supporting the alternate allele and were frequently called uniquely in our callset. To improve downstream analyses, we made the conservative choice of requiring that identified mosaic variants be present jointly in our callset and in the Iossifov or Krumm callsets. This filter greatly improves the precision of our callset (100% for variant presence; Table 1, post-filter) with little change in sensitivity. However, the precision of the classification of the mosaic status of variants remained modest (68%).

**Table 1:** Sanger sequencing validation of variants in the Simons Simplex Collection.

		Chosen	Assay Success	Variant Present	Detection Precision	Variant Mosaic	Classification Precision
Pre-filter	Mosaic	47	37	20	0.54	14	0.37
	Germline *de novo*	50	39	39	1.00	3	0.92
Post-filter	Mosaic	26	19	19	1.00	13	0.68
	Germline *de novo*	50	39	39	1.00	3	0.92

Pre-filter mosaic variants were identified as described in the methods section. Post-filter mosaic variants have the additional requirement that they must be identified jointly in the current callset and one of the callsets produced by Iossifov *et al*. or Krumm *et al*. Assay success refers to technical success of the sequencing assay.

**Fig 1:**
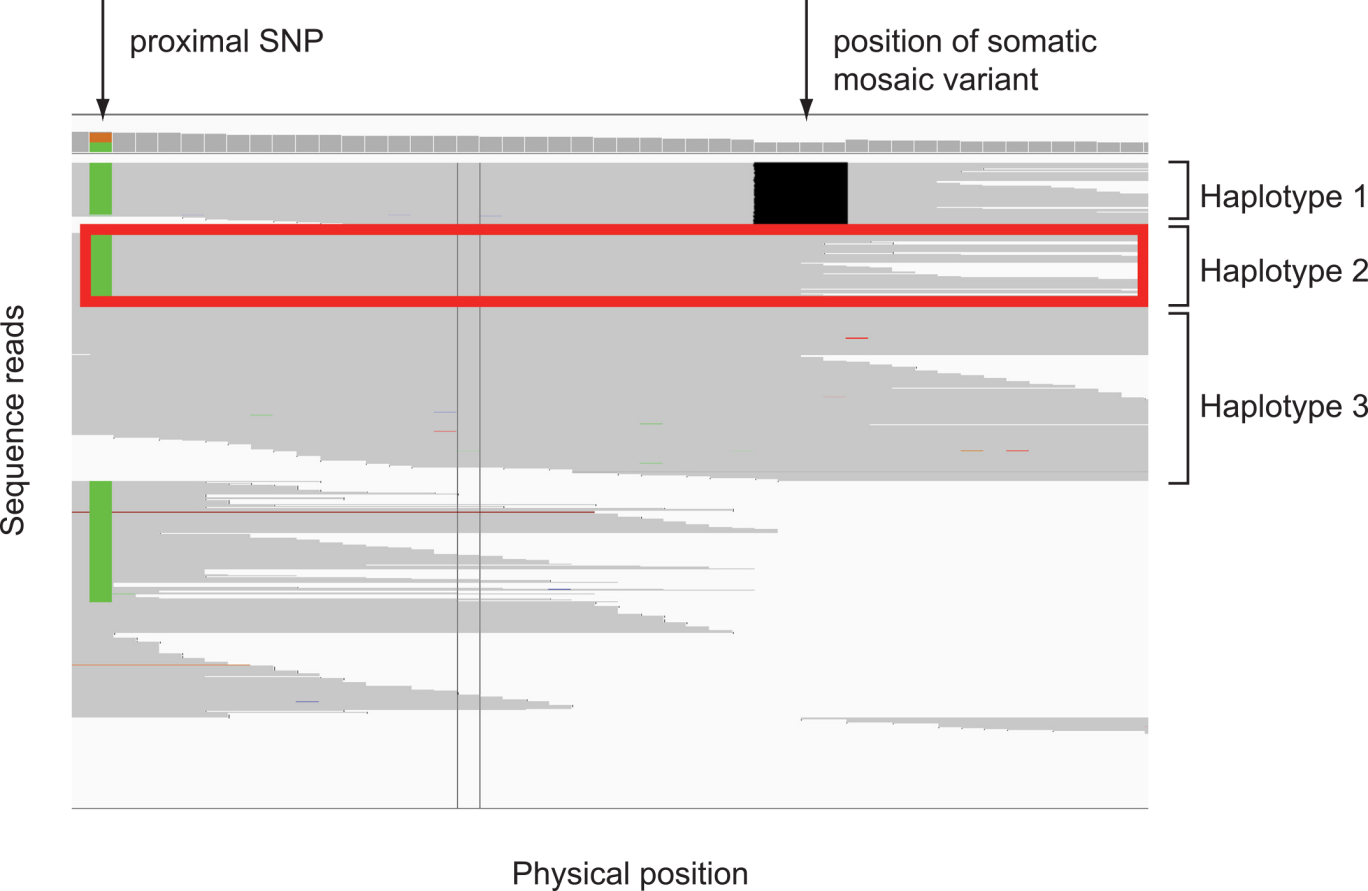
Confirmation of mosaic status by sequence read phasing. Phasing of a *de novo* variant confirms mosaic status of a variant in *OR4M2* in individual 12977.s1. The heterozygous variant on the left is inherited (green pattern; unknown parental origin). The deletion on the right was identified as occurring *de novo*. The presence of multiple reads (box) containing the inherited allele but not the *de novo* variant demonstrates the occurrence of three distinct haplotypes implying post-zygotic origin and mosaicism.

While Sanger sequencing provides an accurate assessment of the presence of *de novo* variants, examination of the chromatograms can provide only approximate estimation of mosaic status. To more accurately assess the mosaic status of the identified variants, we performed sequence read phasing of all identified *de novo* variants. Phasing of the potential mosaic variants relative to nearby inherited heterozygous variants using sequence reads may rigorously confirm the presence of mosaicism. This occurs when three parental haplotypes are inferred: a single haplotype from one parent (e.g. having the minor allele of the neighboring SNP), and two haplotypes from the other parent (e.g. having the major allele of the neighboring SNP) which resolve into a haplotype with the mosaic allele and a distinct haplotype lacking the mosaic allele (Fig 1). We wrote a program called phase-mosaic to perform phasing validation (see Materials and Methods). Of the variants passing filters, phasing was informative for 51 mosaic variants of which 29 were validated as mosaic (57%; Table 2, Pre-filter). Mosaic variants identified by next-generation sequencing that failed phasing confirmation tended to be variants called at high depth with a high AARF. We suspect that these variants appear to be mosaic due to preferential capture of the reference allele during exome enrichment. To correct for this effect, we modified our criteria for the identification of mosaic variants to require that mosaic variants have AARF less than 34%. With these adjusted parameters, the precision of our classification of mosaic status improved to 87% (Table 2, Post-filter).

**Table 2:** Read-backed phasing validation of the mosaic status of identified variants.

		Variants	Phasing Informative	Mosaic	Germline *de novo*	Classification Precision
Pre-filter	Mosaic	342	51	29	22	0.57
	Germline *de novo*	3742	443	27	416	0.94
Post-filter	Mosaic	221	30	26	4	0.87
	Germline *de novo*	3874	468	31	437	0.93

Pre-filter mosaic variants were identified using the binomial test resulting in moderate sensitivity for mosaic status. Post-filter mosaic variants had the added requirement of an AARF of less than 34%, improving classification precision.

Validation of mosaic variants was also performed using pyrosequencing (S11 Table). Likely-gene disrupting (LGD) and missense variants in probands across a range of allele frequencies were chosen for pyrosequencing validation (see Materials and Methods). Consistent with the post-filter results of Sanger sequencing and physical phasing, pyrosequencing validation demonstrated high precision for variant detection and variant classification (Table 3). Of all variants validated by an orthogonal sequencing technology, 16 were validated with multiple validation methods. The results were consistent, except for apparent inaccuracy in the classification of mosaic status by Sanger sequencing.

**Table 3:** Pyrosequencing validation of variants in the Simons Simplex Collection.

	Successful validation	Variant present	Detection precision	Variant mosaic	Classification precision
Mosaic	11	11	1.00	9	0.82
Germline *de novo*	10	10	1.00	1	0.90

**Fig 2:**
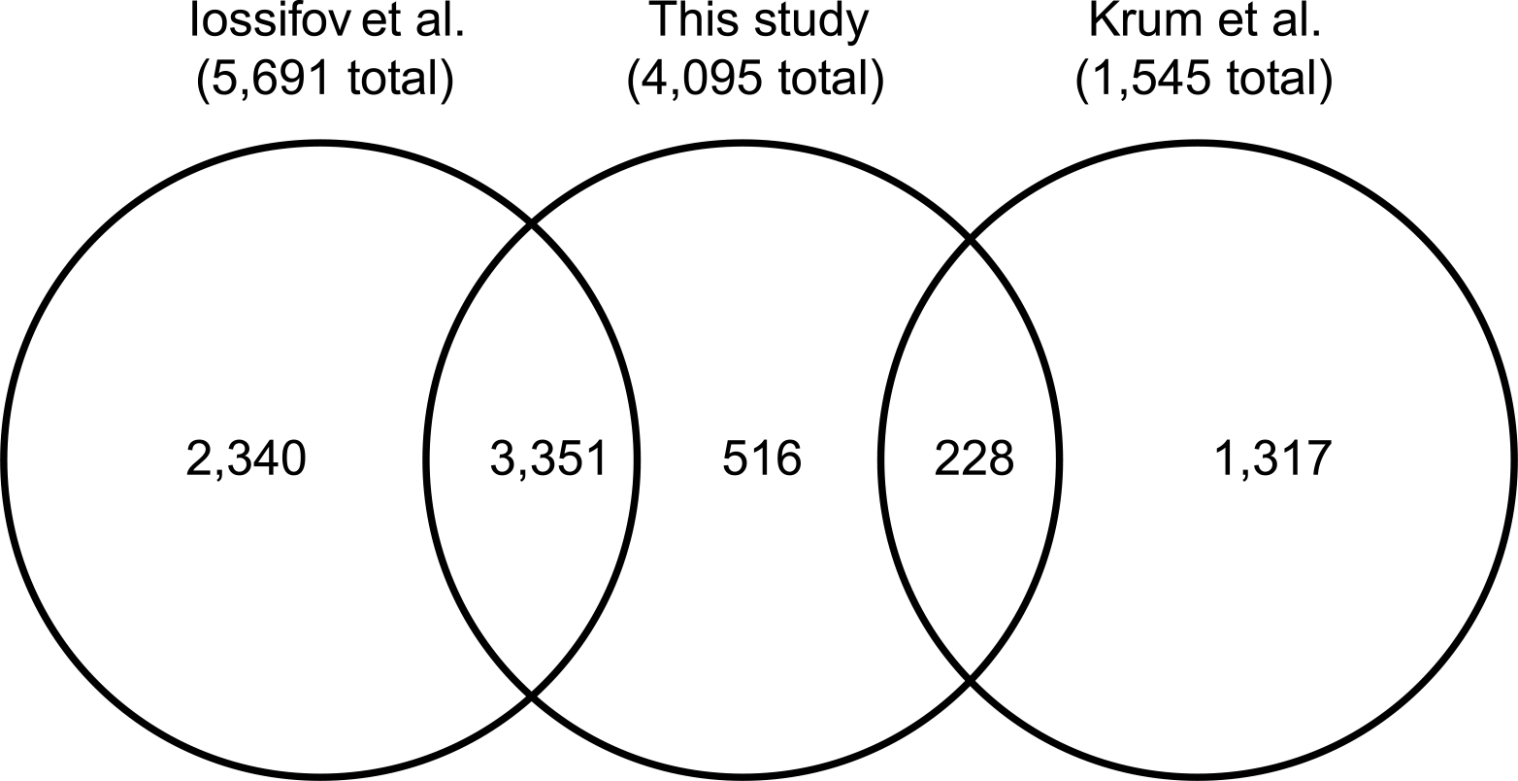
Venn diagram of variants in the high-confidence callset.

After the application of filters, we identified a total of 4,095 *de novo* variants in our high-confidence callset, 221 of which were classified as mosaic. Based on our validation experiments, we estimate that our precision for the presence of the called variants is near 100% with the precision of the classification of mosaic variants measured at 87% or 82% by phasing or pyrosequencing, respectively. Of the variants in our final callset, 3,351 appear jointly in the current callset and the callset produced by Iossifov *et al*. while 228 appear jointly in the current callset and the callset produced by Krumm *et al*. (Fig 2). In the high confidence callset no mosaic mutations were identified that were shared between a sibling pair.

### Properties of mosaic variants

To better understand the properties of variants in our callset, we examined the mutational spectra of the identified mosaic variants relative to germline *de novo* variants (S12 Table). We find that mosaic variants have significantly more deletions than germline *de novo* variants (Fisher’s exact test, p = 5.2e-4). However, the rate of occurrence of other types of mosaic mutations is approximately equal to the rate of occurrence of the corresponding *de novo* mutation. The relative enrichment of mosaic mutations for deletions may indicate an increased rate of false-positive mutation as the identification of indels from next-generation sequence data is known to be difficult. However, our precision when validating mosaic mutation was quite high (see above). We attempted validation of four deletions in the high-confidence callset using Sanger sequencing (S7 Table). All four deletions were found in the sample and three of four were confirmed as mosaic. Besides false-positives, the enrichment of mosaic mutations may indicate a non-reference allele bias, where germline *de novo* deletions are occurring in the samples but are incorrectly classified as mosaic due to mapping errors. Phasing assessed the mosaic status of eight mosaic deletions, six of which were confirmed as mosaic resulting in a classification precision of 75%, slightly less than the overall classification precision of 87% from sequence read phasing. Therefore, inaccurate classification of the mosaic status of *de novo* deletions may contribute to the observed enrichment. An additional hypothesis is that the mechanism underlying mosaic mutation wholly or partly differs from that of germline *de novo* mutation and the relative enrichment of deletions may be attributed to these differences in underlying mechanisms.

### Rates of mosaic mutation

**Fig 3:**
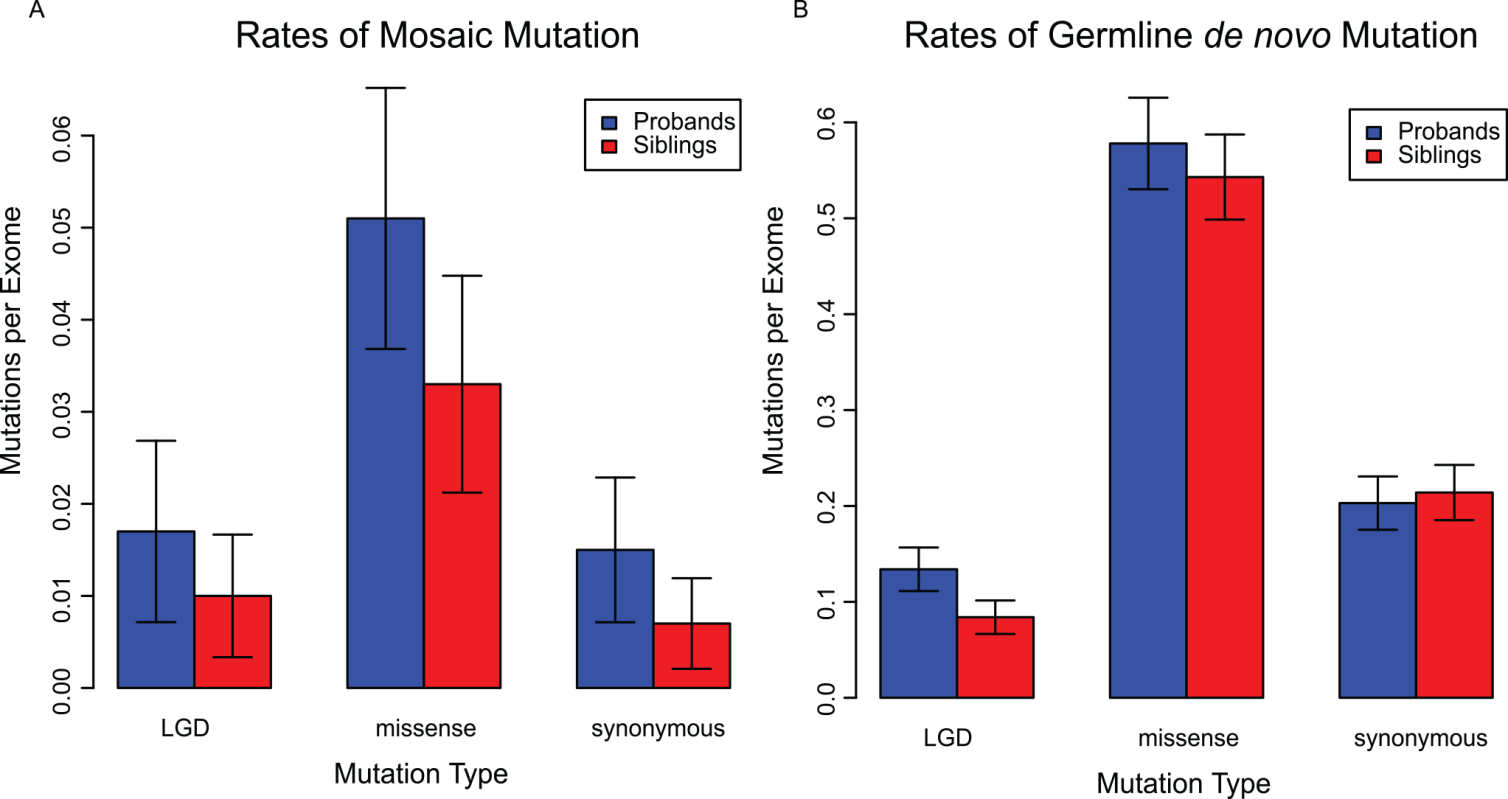
Rates of mutation in the Simons Simplex Collection. Average number of mutations per exome, as calculated using joint 40x regions. Error bars represent the 95% confidence interval for the mean. (A) Mutations categorized as mosaic with AARF < 0.34 and q < 0.05. (B) Germline *de novo* mutations as determined by q > 0.05 or AARF > 0.34.

Previous studies have indicated that *de novo* mutations occur at higher rates in probands relative to controls leading to the implication of *de novo* variants as contributing to disease diagnoses [13,17]. We utilized our high-confidence mosaic variant callset to compare the rates of mosaic and germline *de novo* mutation in probands relative to unaffected siblings. Following the protocol of Iossifov *et al*. we defined regions of joint 40x coverage in children of quad families and extrapolated rates of mutation within these joint 40x regions to the entire capture region (Fig 3; S13 Table). Consistent with previous results, we find that germline *de novo* LGD mutations are significantly enriched in probands relative to controls (p = 0.001). In addition we find that all classes of mosaic mutations are significantly enriched in probands (p = 0.003). Interestingly, we observe contribution to disease from all classes of mosaic variation, whereas the contribution of germline *de novo* variation to disease is primarily from LGD mutations.

**Fig 4:**
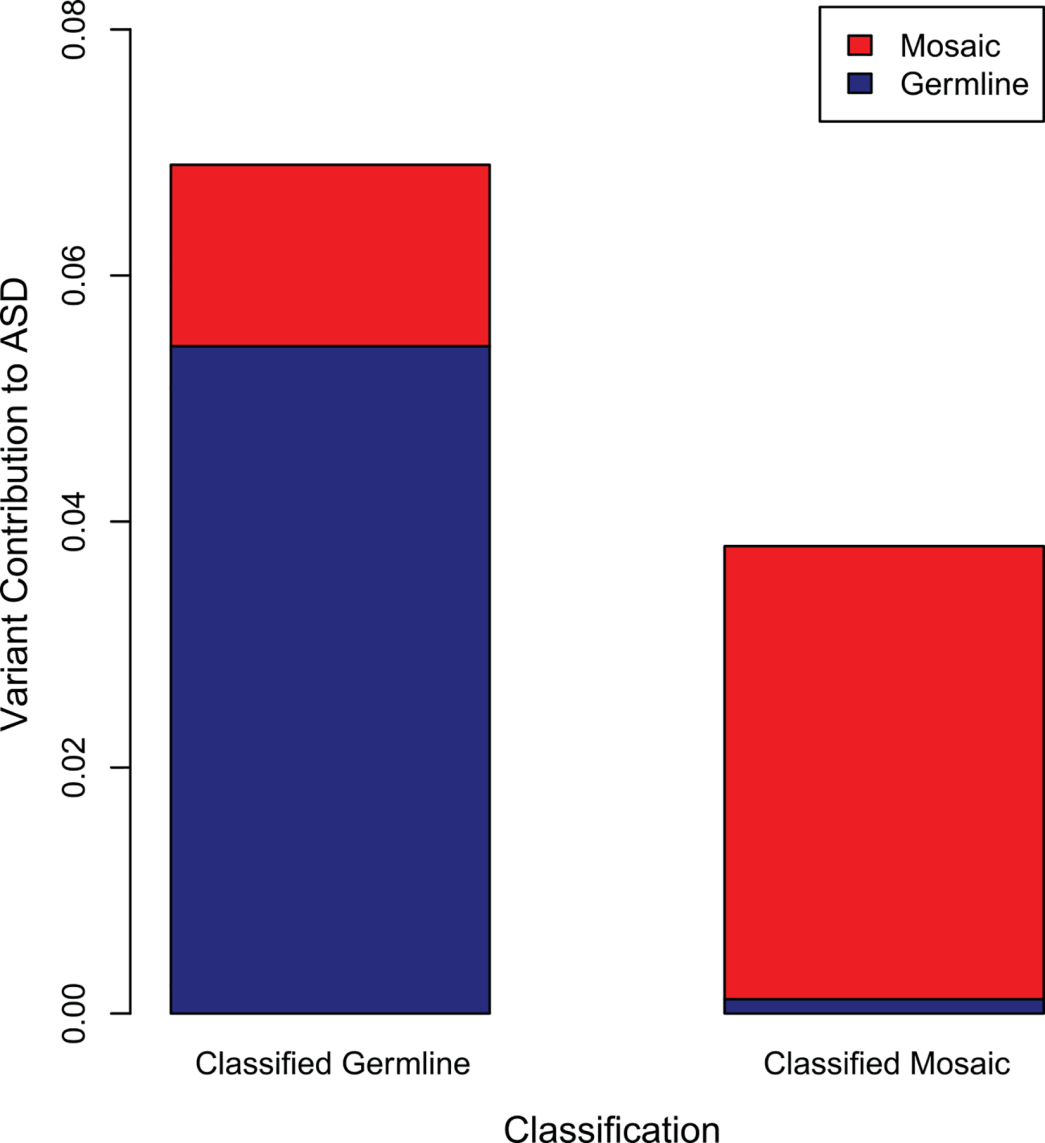
The Contribution of *de novo* Mutations to ASD. The contribution of classified mosaic and germline mutations are shown. For each classification, the contribution is divided into correctly classified and incorrectly classified variation. The contribution of incorrectly classified mosaic variants (called germline; left bar, upper region) is substantial, but the contribution of incorrectly classified germline variation (called mosaic; right bar, lower region) is small.

To account for errors in the classification of variants as either mosaic or germline *de novo*, we extended our model of contributory variation to include incorrectly classified variants. In this model, mosaic variants incorrectly classified as germline *de novo* account for a substantial portion of the genetic contribution of variants classified as germline *de novo* (Fig 4). In total, mosaic variation contributes to 5.1% of ASD cases (95% credible interval [CI], 1.3% to 8.9%) while all classes of germline *de novo* variation contribute to 5.6% of ASD cases (95% CI, 1.8% to 9.4%). The percent of contributory variants to total variants are measured as 6.0% (95% CI, 2.0% to 10%) and 33% (95% CI, 9.6% to 54%) for germline *de novo* and mosaic variants, respectively.

### Functional consequences of *de novo* mutation in the SSC

While differences in the rates of mutation in affected individuals may implicate mutations in disease, it may also be the case that mutations in probands occur in more functionally conserved genomic regions. Using all of the mutations in our high-confidence callset, we test the hypothesis that mutations in probands occur at more conserved genomic regions. For this analysis, we use three measures of conservation: base-level conservation as measured by PhyloP, and gene-level conservation as measured by HomoloGene or ExAC (S14 Table) [29–31]. The gene-level conservation measures from ExAC and Homologene are complimentary as HomoloGene provides a measure of evolutionary conservation while ExAC provides a measure of conservation in extant human populations. We find that germline *de novo* LGD mutations occur at more highly conserved positions in probands relative to controls as measured by PhyloP score (p = 0.048, effect = 0.54). For mosaic missense mutations, we observe a stronger effect (0.91), although the test does not reach statistical significance due to the small sample size (p = 0.179). We find that germline *de novo* missense variants occur significantly more often in genes thought to be intolerant of loss-of-function mutation as annotated by ExAC (p = 0.013). While our analysis does not show that germline *de novo* missense mutations occur at significantly higher rates in probands relative to siblings (p = 0.30), germline *de novo* missense variants likely target genes less tolerant of functional mutation more frequently in affected individuals relative to their siblings.

The initial publication of the SSC exome sequencing data demonstrated enrichment of mutations in specific classes of gene targets [13]. To find insight into the mutational mechanisms and functional consequences of mosaic mutation, we replicated this analysis (with modification) using our high-confidence callset. This analysis confirmed the significant enrichment of germline *de novo* LGD mutations from probands in FMRP targets, chromatin modifiers, and genes with known LGD mutations in intellectual disability or schizophrenia (S15 Table). In addition, we observed enrichment of mosaic missense and LGD mutations in probands and siblings in genes involved in embryonic development (18 observed versus 12.6 expected for probands; 9 observed versus 6.7 expected for siblings), however this enrichment did not reach statistical significance (p = 0.12 for probands; p = 0.30 for siblings). We also tested for overlap between genes targeted by mosaic missense and LGD mutations and a set of 107 genes that had been strongly implicated in ASD using the null-length model (see Methods) [32]. We found three of the 98 genes with mosaic missense or LGD mutations in probands have been previously implicated in ASD (*KMT2C*, *NCKAP1*, and *MYH10*). The presence of the *NCKAP1* was confirmed by Sanger sequencing, but did not confirm its mosaic status. However, the number of mosaic mutations in ASD genes does not reach statistical significance for enrichment in the set of 107 ASD genes (3 observed; 1.15 expected; p = 0.109). Zero of 52 genes targeted by mosaic missense or mosaic LGD mutations in siblings were previously implicated in ASD.

## Discussion

There are three major conclusions from this study. First, we show that mosaic mutations occur frequently in individuals diagnosed with ASD and their unaffected siblings. We identify a total of 4,095 *de novo* mutations, of which 221 (5.4%) are classified as mosaic. This is similar to previously reported estimates for the fraction of mosaic variants in individuals with intellectual disability [21]. In light of previous work demonstrating the presence of mosaic mutation in diverse body tissues we believe that the mosaic mutations we identified are not unique to blood but are dispersed throughout the body [18]. Although the early steps of our pipeline were performed explicitly to increase our sensitivity for mosaic mutation, many of our filtering steps were conservative and we likely underestimate the true fraction of mosaic mutations in the Simons Simplex Collection. Our filtering approach combined with recent improvements in variant detection algorithms likely accounts for most of the differences between our variant callset and the callsets published by Iossifov *et al*. and Krumm *et al*. [13,15]

Second, we find that mosaic mutations are significantly enriched in probands relative to their siblings. Using our model of contributory variation we estimate that 33% of mosaic mutations contribute to 5.1% of ASD diagnoses. As mosaic mutations arise post-zygotically in only a fraction of the cells of an individual, we expect that these results have implications for the interpretation of twin studies, especially observed cases of phenotypic discordance between monozygotic twins.

Third, we find that tissue-specific mosaic mutations do not occur in the paired samples at our limit of detection. Given the lack of publications on validated tissue-specific mutations in tissues without visual abnormality and the absence of brain-specific mutation in a centenarian [24], we do not believe that this finding is unexpected. While a recent study reported tissue-specific mosaic mutation [33], the results presented here include validation of detected mutations showing that, in our study, these were false positive findings. It is possible that tissue-specific mutations do contribute to ASD in at least some cases. However, discovery of such variation and its implication in disease may require larger numbers of samples or more sensitive approaches (such as single-cell sequencing).

Together, these results indicate that mosaic mutations are an identifiable subset of *de novo* mutation. As heritable factors that may arise in a single twin of a monozygotic pair [19,20], contributory mosaic mutation implies some expected level of discordance between monozygotic twins due to heritable factors arising post-zygotically. Furthermore, high-confidence identification of contributory mosaic mutation in affected probands implies a lower risk of familial recurrence in some families.

## Materials and Methods

### Paired sample whole-exome sequencing

Paired samples were obtained from the University of Maryland Brain and Tissue Bank as detailed in S1 Table. Individuals were diagnosed with ASD (n = 12) or were controls; criteria for diagnosing ASD included the Autism Diagnostic Interview-Revised (ADI-R), Childhood Autism Rating Scale (CARS), and Autism Diagnostic Observation Schedule (ADOS) as detailed S1 Table. DNA was extracted from tissue dissections according to protocols in the QIAGEN Genomic DNA Handbook. Exonic regions were selectively captured using Agilent SureSelectXT Human All Exon V5. Sequencing was performed at the Center for Inherited Disease Research at Johns Hopkins generating 100 bp sequence reads on an Illumina HiSeq. CIDRSeqSuite version 3.0.1 was used for processing of the raw data files. BCL files were converted to qseq format using Illumina’s BCL converter. qseq files were then demultiplexed and converted to FASTQ files using a custom demultiplexer. Paired-end alignment was performed using BWA aln to the 1000 genomes hg19/GRCh37 reference genome [34]. SAM files were sorted, converted to BAM, and duplicates were marked with Picard. GATK was used for local realignment and base quality score recalibration [35,36]. Quality metrics for these data are provided in S2 Table.

### Paired sample tissue-specific variant calling

Tissue-specific variants were called from paired samples using MuTect 2.7–1 for SNV discovery and Strelka 1.0.13 for indel discovery [22,23]. Input to these programs requires specifying a “tumor” and a “normal” sample. For each paired sample, variants were called twice so that mosaic variants were identified in both the brain and heart/kidney tissue. Validation of these variant calls was performed as indicated in S5 Table (Targeted Sequencing 1), with variants with the most severe functional effect selected for validation.

To more carefully examine the properties of the mosaic variants called by MuTect, variants were recalled jointly in all samples using the GATK’s HaplotypeCaller in the “GENOTYPE_GIVEN_ALLELES” mode [35,36]. These variant calls were converted to a text based file format (S3 Table) and allelic noise was annotated as an additional quality metric. Allelic noise was measured as the fraction of reads supporting the alternate allele relative to the total number of reads in all samples genotyped as homozygous for the reference allele by the HaplotypeCaller. Samples with called somatic variants were excluded from the calculations of allelic noise. If multiple alternate alleles were present, only the highest alleleic noise was recorded. Using allelic noise and the quality metrics annotated by the GATK’s HaplotypeCaller, the overall quality of the variants was assessed manually. Validation of the highest quality variants was attempted and the results of the validation are shown in S5 Table (Targeted Sequencing 2) and S6 Table.

For visualization, the properties of variants including "BaseQRankSum", "FS", "MQ", "MQRankSum", "ReadPosRankSum" and "SOR" were examined in the Illumina Platinum Genomes (see below) and variants called by MuTect. These properties were collectively scaled, principal components analysis was performed and the original variant features were transformed into the principal components. The variants were then plotted along these principal components as shown in S1 Fig.

### *In silico* mixing experiment using NA12878 and NA12882

200x sequence data from NA12878 and NA12882 were downloaded from EBI (ERP001775). The specific runs chosen were ERR174324, ERR174325, ERR174326, ERR174327, ERR174328, ERR174329, ERR174330, ERR174331, ERR174332, ERR174333, ERR174334, ERR174335, ERR174336, ERR174337, and ERR174338 for NA12878 and ERR174347, ERR174348, ERR174349, ERR174350, ERR174351, ERR174368, ERR174369, ERR174370, ERR174371, ERR174372, ERR174373, ERR174374, ERR174375, ERR174376, and ERR174377 for NA12882. Sequence data were aligned to the 1000 Genomes phase 2 human reference genome using BWA MEM version 0.7.9a and aligned sequence reads were sorted using SAMtools [34,37]. Aligned sequence data were then combined into single files and *in silico* mixing with subsampling was performed using submixbam (https://github.com/DonFreed/submixbam) version 290fda over GIAB high-confidence regions (http://ftp-trace.ncbi.nih.gov/giab/ftp/data/NA12878/variant_calls/GIAB_integration/union13callableMQonlymerged_addcert_nouncert_excludesimplerep_excludesegdups_excludedecoy_excludeRepSeqSTRs_noCNVs_v2.19_2mindatasets_5minYesNoRatio_AddRTGPlatGenConf_filtNISTclustergt9_RemNISTfilt_RemPartComp_RemRep_RemPartComp_v0.2.bed.gz; accessed Oct 14^th^ 2015; currently available from ftp://ftp-trace.ncbi.nih.gov/giab/ftp/data/NA12878/analysis/GIAB_integration/). A variant calling and assessment pipeline was written using Snakemake [38]. This pipeline called variants from mixtures using the GATK’s HaplotypeCaller version 3.4–46. The sensitivity of the HaplotypeCaller for variants known to be present in NA12878 and absent from NA12882 was then evaluated using hap.py (https://github.com/Illumina/hap.py). Scripts are available in S1 Code.

### Simons Simplex Collection analysis

Analysis of the data in the Simons Simplex Collection made use of cloud computing via Amazon Web Services (AWS) for variant calling (GATK HaplotypeCaller), merging gVCFs (GATK MergeGVCFs) and genotyping (GATK GenotypeGVCFs). Starcluster (http://star.mit.edu/cluster/) was used for deployment and configuration of clusters of virtual machines on AWS Elastic Cloud Compute (EC2) and a customized Amazon Machine Image (AMI) was created containing GATK 3.5–0, samtools 1.2, Python 3.5.1, and nda_aws_token_generator version 20b72 (https://github.com/NDAR/nda_aws_token_generator) [35–37]. c3.xlarge, r3.xlarge and r3.2xlarge instances were used for variant calling, merging gVCFs and genotyping, respectively. During variant calling and merging, the available disk space on each node was used as a complex resource to aid in job allocation. With c3.xlarge instances, ephemeral storage partitions were combined into single logical volumes using RAID 0. During genotyping, node ephemeral disk partitions were combined into a single network attached storage volume using GlusterFS (https://www.gluster.org/).

### Simons Simplex Collection variant discovery

Aligned whole-exome sequence data from 8,950 individuals in the SSC was accessed through the National Database for Autism Research (NDAR) on Amazon Web Services Simple Storage Service (AWS S3) (https://ndar.nih.gov/study.html?id=334). We excluded 16 individuals from families 11366, 11368, 11377 and 11380 due to data processing issues. Variants were called using the GATK (v. 3.5–0) HaplotypeCaller in gVCF mode with standard variant annotations and additional arguments -ploidy 5, -A GCContent and–A AlleleBalance over NimbleGen EZ-SeqCap v2.0 targets with 50 bp of padding [35,36]. gVCFs of 20 families were combined using GATK MergeGVCFs resulting in 120 merged gVCF files. All gVCF files were genotyped across capture regions in parallel using the GATK GenotypeGVCFs command with the arguments -stand_call_conf 25.0, -stand_emit_conf 20.0 along with the arguments used with the HaplotypeCaller as described above. The genotyping step had high memory requirements over some target regions, causing some jobs to fail even with 116 GB of memory allocated to the java virtual machine. Failed capture regions were repeated with the additional argument—max_alternate_alleles 5. However, we excluded 53 capture targets due to persistent memory errors (S16 Table). These capture targets were highly enriched for overlap with known simple repeats (UCSC Simple Repeats Track in BED format; tested using BEDtools fisher; Fisher’s Exact Test, p < 0.00001). Variant calls over each capture target were then concatenated and duplicate calls due to overlapping padded targets were removed.

### Simons Simplex Collection variant filtration

In addition to the variant annotations produced by the GATK, raw variants were annotated with the number of sequencing reads supporting the reference allele relative to total number of sequence reads. This information was added to the VCF’s INFO field as the annotation “AbHetUser”. Variants were filtered using the GATK variant quality score recalibration pipeline. The recommended parameters for whole-exome sequencing were used minus the–an QD parameter and with the additional parameter–an AbHetUser. These parameters were chosen for their superior sensitivity and specificity for validated *de novo* variants in the SSC (S17 Table). SNPs were filtered with a sensitivity tranche of 99.3% while indels were filtered with a sensitivity tranche of 98%.

### Simons Simplex Collection *de novo* and mosaic variant identification

*De novo* variants were identified using the tool find_denovo, a tool we wrote in the C programing language, with default parameters (https://github.com/DonFreed/find_denovo). find_denovo identifies alleles which are present in children but absent from their parents. It then applies a number of filters including a minimum number of reads for all trio members (20), a minimum number of reads supporting the alternate allele in the child (3), a minimum phred-scaled confidence for the presence of the *de novo* allele in the child (20) and the absence of the *de novo* allele in the parents (20), and a maximum number of individuals genotyped for the allele in the cohort (2). *De novo* variant effects were then annotated using SnpEff [28]. Families 11060, 11431, 11628, 11714, 11905, 12173, 12230, 12401, 12456, 12809, 12879, 13143, 13949, 14025, and 14355 were excluded as more than 10 *de novo* mutations were observed in at least one child in the family.

Mosaic variants were identified from *de novo* variants using the binomial test to examine the alternative hypothesis that the *de novo* allele is supported by significantly fewer sequence reads than expected from the read depth. We use p = 0.5 as the expected fraction of sequence reads supporting the *de novo* allele. p-values were adjusted using the Benjamini-Hochberg procedure with a FDR of 0.05 and variants with q < 0.05 were called mosaic. In the final callset we add the requirement that mosaic variants must have an AARF of less than 34%. In addition, mosaic variants that were identified uniquely in our callset and not in the callsets produced by Iossifov *et al*. or Krumm *et al*. were filtered.

### Phasing of variants in the Simons Simplex Collection

Variants identified as *de novo* in the Simons Simplex Collection were phased to nearby inherited variants to validate mosaic status and to determine the parental haplotype of the variant allele using phase-mosaic, a tool we wrote in Java and Python (https://bitbucket.org/donald_freed/phase-mosaic, version f47bcd). For each identified *de novo* variant, sequence data 500bp upstream and downstream of the variant was downloaded to the local machine from AWS Simple Storage Service (S3) for each member of the pedigree. Variants were then recalled using the GATK version 3.5–0 compiled with the VariantReadIds annotation. Phasing was then performed on the resulting VCF files.

### Rates of mutation in the Simons Simplex Collection

Regions of 40x coverage were defined for each individual in quad families using BEDtools genomecov–bga with the resulting BedGraph file converted to a BED file using a custom script (S1 Code) [39]. BEDtools was then used to intersect the 40x BED file for each member of a trio and the target capture file to produce a joint 40x BED file for the trio. Variants in the callset were annotated based on their presence or absence in the joint 40x region using custom scripts (S1 Code). The length of the genome present in the joint 40x region was recorded for each child in a quad family. Finally, the rate of *de novo* mutation for each individual and each class of mutation was calculated from the size of the joint 40x region and the number of mutations in joint regions identified in the child. These rates were then extrapolated to the entire capture region. The mean and standard deviation of the rates observed in probands and siblings are reported in S13 Table.

Iossifov et al. previously reported a model of *de novo* variation in which siblings have a baseline rate of *de novo* mutation while probands have the same baseline rate and additional mutation due to their affected status [13]. We expand this model to distinguish between germline *de novo* and mosaic variation while incorporating errors in classification of mosaic status. In siblings the observed rate of mosaic or germline *de novo* variation was modeled as the sum of correctly and incorrectly classified baseline variation. In probands the models included correctly and incorrectly classified contributory variation in addition to the baseline variation. Classification error rates were for siblings modeled as either incorrectly classified baseline variation over correctly and incorrectly classified baseline variation. For probands, classification error rates were modeled as incorrectly classified baseline and contributory variation over correctly and incorrectly classified baseline and contributory variation.

These models were solved to obtain the rate and fraction of contributory variation using the observed rates of mutation and classification errors as measured by phasing validation. Classification error rates were calculated separately for probands and siblings and for germline *de novo* and mosaic classification. Uncertainty in classification error rates was modeled using the beta-binomial distribution with phasing validation results as model parameters. A 95% credible interval was obtained through 10,000 permutations with classification error rates obtained by random draws from their respective distributions.

### Simons Simplex Collection variant conservation

Variants were annotated with PhyloP conservation score, taxonomic conservation as reported by NCBI’s HomoloGene database, and the probability of null mutations being deleterious (“pNull”) as reported by ExAC [29–31]. BigWig files containing genome-wide PhyloP scores were downloaded from UCSC (ftp://hgdownload.cse.ucsc.edu/goldenPath/hg19/phyloP100way/hg19.100way.phyloP100way.bw; accessed Nov. 10^th^, 2015) and were used to annotate variant conservation. Gene-level conservation was annotated by querying the NCBI’s HomoloGene database using Biopython and Entrez to find the earliest taxonomic unit reported to sharing the gene containing the mutation [40]. These taxonomic units were then converted to numeric scores where 0 corresponds to conserved in Homo while 31 corresponds to conserved to the root of the HomoloGene taxonomic tree. ExAC gene summary data were downloaded from (ftp://ftp.broadinstitute.org/pub/ExAC_release/release0.3/functional_gene_constraint/README_fordist_cleaned_nonpsych_z_data_pLI_2016_01_13.txt; accessed Feb. 11^th^ 2016) and variants were annotated with the reported probability of their respective gene being intolerant of loss-of-function mutation. Using these data, mutations present in probands were compared to mutations present in siblings with the Wilcoxon rank sum test. The results are reported in S14 Table.

### Gene target overlap and recurrence

Methods for analysis of gene target overlaps and recurrence were adopted, with modification, from Iossifov *et al*. [13]. RefSeq genes were downloaded from the UCSC Table Browser (https://genome.ucsc.edu/cgi-bin/hgTables; accessed Jul. 16^th^ 2015). The “chr” prefix was removed from the chromosome names and the raw table was sorted by chromosome and position. Coordinates of coding sequence starts and stops were extracted from the RefSeq table in BED format using custom scripts (S1 Code) and overlapping coding sequences were merged using BEDtools [39]. This file was intersected with the BED file of the target capture region and the length of each gene in the target region was calculated. These data were then combined with data of gene membership in gene sets from S7 Table of Iossifov *et al*. and the high-quality callset to produce a table describing the number of observed mutations in each gene and each gene’s set membership [13].

Given their observed contribution to ASD diagnosis (S13 Table), only mosaic missense and germline *de novo* LGD mutations were analyzed and these mutations were analyzed in both probands and siblings. These analyses were performed using a null length model where the probability of a mutation occurring within a gene is proportional to its length targeted for exome capture relative to the total size of the capture target. For every mutation-type, individual combination, we calculate the following: (1) The expected number of recurrent mutations and a p-value for the observed number of recurrent mutations from 10,000 simulations using sampling with replacement. (2) For each gene set from Iossifov *et al*. we calculate the expected number of genes harboring mutation present in the gene set, given the length of capture targets of genes within the set relative to the total length of all gene capture targets. Using a two-sided binomial test, we test for observed enrichment or depletion from the expectation based on the null length model.

For testing the enrichment of mosaic missense and LGD mutations in genes implicated in ASD, we used the approach described above with the target gene set of 107 candidate genes identified by De Rubeis *et al*. [32].

### Pyrosequencing

Amplification and sequencing primers were designed for all loci using PyroMark software and the NCBI’s Primer-BLAST [41]. Additionally, primers were checked for overlap with common SNPs using the UCSC Genome Browser [42]. Samples were amplified according to protocols in the Qiagen Pyromark PCR kit with a single biotinylated primer. Pyrosequencing was performed and data were analyzed by the Johns Hopkins Genetic Resources Core Facility.

### Amplicon-targeted sequencing

Sequence libraries were generated from purified DNA according the Nextera XT DNA Library Preparation Guide. Sequence data were then generated on an Illumina MiSeq using a MiSeq Reagent Kit v2. Sequence reads were aligned to the human reference genome (UCSC hg19) using BWA and reads supporting the reference or alternate alleles were counted [34].

### Sanger sequencing

Mosaic variants and germline *de novo* variants for validation were chosen at random from variants present in samples on hand. In total 97 variants were chosen for validation, 50 germline *de novo* variants and 47 mosaic variants. Primers for polymerase chain reaction amplification were designed using Primer-BLAST [41]. Amplification was performed using DNA isolated from whole blood and Sanger sequencing was performed at the Johns Hopkins University School of Medicine Synthesis and Sequencing Facility.

### Accession numbers

For paired samples from the University of Maryland Brain and Tissue Bank, BAM files and corresponding phenotypic data are available from the Database of Genotypes and Phenotypes (dbGaP) at the National Institutes of Health (phs000337). The 50x Illumina Platinum Genomes dataset is publicly available from EBI with accession ERP001960 and ERP002490. All runs in ERP001960 were used while select runs were used from ERP002490 (see Materials and Methods). Sequence data from the Simons Simplex Collection were obtained via controlled access through the National Database for Autism Research (http://ndar.nih.gov/study.html?id=334).

## Supporting Information

S1 Fig Quality metrics of variants GIAB variants and MuTect calls. (A) Principal components of scaled variant features in the GIAB callset visualized by log-transformed density (blue) overlaid with variants called by MuTect (red). Much of the density of GIAB variants was clustered around (0,0), with the MuTect variants having significantly more spread. 96.0% of GIAB variants cluster within a Euclidian distance of three from the origin, as did only 41.5% of variants called by MuTect. (B) Noise measurement in the GIAB and MuTect variants (log-scale). MuTect variants were called at sites with drastically more noise. While only 0.3% of GIAB variants had an allelic noise above 0.01, 60.2% of MuTect variants did.
(EPS)

S2 Fig Performance evaluation of submixbam. (A and B) Evaluation of subsampling functionality of submixbam. Target depths are consistent with measured depth across a range of target depths. (C and D) Evaluation of mixing functionality of submixbam. submixbam performed highly precise and accurate mixing and subsampling.
(EPS)

S3 Fig Sensitivity of the GATK HaplotypeCaller for mosaic variation. (A) Sensitivity of the HaplotypeCaller for simulated mosaic variants from real sequence data at a sequencing read depth of 50x using various ploidy arguments. Higher ploidies resulted in higher sensitivity. (B) Comparison of ploidy 2 and ploidy 5 at 30x and 50x depth. Higher depths and high levels of the ploidy argument resulted in increased sensitivity.
(EPS)

S4 Fig Overview of the pipeline for calling variants in the Simons Simplex Collection. For each step the total size of compressed data is given (*e*.*g*. 20 TB), number of individuals or files, analysis tool (*e*.*g*. Genome Analysis ToolKit [GATK] argument), average runtime job, and Amazon Web Services (AWS) EC2 instance type used in the analysis (*e*.*g*. c3.xlarge). Abbreviations: gVCF, genomic Variant Call Format file; glusterFS, a scalable network file system; NAS, network attached storage. Analyses were performed using AWS except the variant concatenation which was performed on a local cluster.
(EPS)

S1 Table Tissue samples obtained from the University of Maryland Brain and Tissue Bank. PMI, postmortem interval.
(XLSX)

S2 Table Quality control metrics of whole-exome sequence data from paired samples as reported by CIDR. TARGET_TERRITORY is the size of the exome-capture target. PCT_PF_UQ_READS_ALIGNED is the number of unique reads passing vendor quality filters that were successfully aligned to the genome. MEAN_TARGET_COVERAGE is the mean coverage over exome-capture targets. ZERO_CVG_TARGETS_PCT is the fraction of targets without coverage over any base.
(XLSX)

S3 Table Tissue-specific mosaic SNVs identified by MuTect. Quality metrics for each mutation are shown (n = 284). The “Called” column indicates samples in which the mutation was identified by a germline variant caller. For each mutation, the total number of sequence reads (DP) and the number of reads supporting the alternate allele (AD) are reported for each individual. Attempted validation of variants by pyrosequencing (blue), targeted sequencing (orange), or both pyrosequencing and targeted sequencing (green) are indicated. Samples from brain (B), heart (H), or kidney (K) are noted as in S1 Table.
(XLSX)

S4 Table Tissue-specific mosaic indels identified by Strelka. Attempted validation of variants by targeted sequencing (orange) is indicated.
(XLSX)

S5 Table Amplicon-targeted sequencing of potential tissue-specific variants in paired samples. (XLSX)

S6 Table Pyrosequencing of potential tissue-specific variants in paired samples. (XLSX)

S7 Table All *de novo* variants identified in the Simons Simplex Collection. Gene_id, refseq_id and functional annotations were converted from the SnpEff annotation of a VCF. p_mosaic is the p-value from a binomial test of the probability of seeing the observed number of reads supporting the alternate allele. q_value is the p-value corrected for false-discovery using the Benjamini-Hochberg procedure. gene_conservation is a measure of gene-level conservation from HomoloGene (see Methods). PhyloP_score is the reported PhyloP conservation score at the variant position. sanger_validation records whether the variant was selected for validation using Sanger sequencing. sanger_technical records whether Sanger sequencing was technically successful and sanger_result records the interpretation of the chromatogram. exclude indicates the variant occurs in a sample that was excluded due to a high number of observed *de novo* variants. p_recessive and p_null are annotated from the ExAC data file. joint_40x indicates the variant occurs with the joint 40x region for that individual. phase_mosaic indicates that the variant could be phased to a nearby inherited variant and whether phasing indicates the variant is mosaic (True or False). If phasing indicates the variant is mosaic, mosaic_frac indicates the expected fraction of cells harboring the mosaic variant according to the phasing analysis. parent indicates the parental haplotype on which the variant occurs. pyro_validation indicates that the variants was selected for pyrosequencing and pyro_result indicates the result of pyrosequencing. in_final_callset indicates that the variant was included in the high-confidence callset of 4,095 variants. 6,408 variants are shown.
(XLSX)

S8 Table Summary of the relationship between the Krumm/Iossifov callsets and the current callset. Filters are applied from left to right. The VQSR column shows the number of variants filtered by the GATK’s Variant Quality Score Recalibration pipeline. The columns called_in_fa and called_in_mo indicate that the variant was genotyped as present in the father or mother, respectively. The column child_alt_dp indicates that fewer than three sequence reads passing quality filters were observed to support the alternate allele in the child. The columns child_dp, fa_dp, and mo_dp indicate a depth of less than 20 sequence reads were observed in the child, father, or mother, respectively. The columns child_PL, fa_PL, and mo_PL indicate a phred-scaled likelihood for the presence of the genotype (in the child) or the absence of the genotype (in the parents) of less than 20. The column multi_individuals shows the number of variants filtered due to their presence in more than two individuals.
(XLSX)

S9 Table Variants in the Simons Simplex Collection as identified by Iossifov *et al*. [13]. Columns 1–4 display variant information. Column 5 shows the name of the individual harboring the variant. Column 6 indicates if the variant was reported as validated by Iossifov *et al*. Column 7 displays the reason the variant was not called in the current study; see the legend of S8 Table for more information. ‘-‘ indicates the variant was called by this study. If the variant was filtered by the VQSR, the VQSLOD is reported. If the variant was genotyped as present in more than two individuals, the individuals genotyped for the variant are reported.
(XLSX)

S10 Table Variants in the Simons Simplex Collection as identified by Krumm et al. [15]. See the legend of S9 Table. Column 6 indicates the validation status of the variant as reported by Krumm *et al*. ND indicates that the variant was not chosen for validation, Y indicates that the variant was successfully validated, N indicates that validation was successfully performed, but the variant was not identified by Krumm *et al*.
(XLSX)

S11 Table Pyrosequencing validation of *de novo* variants in the Simons Simplex Collection. (XLSX)

S12 Table The mutation spectra of mosaic variants relative to germline *de novo* variants. Odds ratios of less than 1 indicate mosaic variants are relatively depleted for those events while odds ratios of greater than 1 indicate relative enrichment. p values were calculated using a Fisher’s exact test.
(XLSX)

S13 Table Rates of *de novo* mutation in individuals in the Simons Simplex Collection. Rates of mutation were measured using joint 40x regions in probands and siblings in quad families as described in the Methods section.
(XLSX)

S14 Table Conservation at sites of *de novo* variation in probands and siblings. Each variant was annotated with measures of conservation as described in the Methods section. Measures of conservation were compared between probands and unaffected siblings and p values were calculated using a Wilcoxon rank sum test. Estimates of effect size were calculated as the estimated difference in ranks.
(XLSX)

S15 Table Gene target overlap and recurrence analysis. Column A describes mutation type and individual classes used in the analysis. Columns B through G describe the results of the recurrence analysis. Columns H though O describe gene target enrichment analysis.
(XLSX)

S16 Table Capture targets excluded due to extremely high memory usage. (XLSX)

S17 Table Variant quality score recalibration sensitivity tranches. Sensitivity and specificity are calculated using variants reported as validated by Krumm *et al*. and Iossifov *et al*. or failing validation by Krumm *et al*. [13,15].
(XLSX)

S1 Code Code used in the described analyses. (GZ)
